# SGK-1 mediated inhibition of iron import is a determinant of lifespan in *C. elegans*

**DOI:** 10.17912/micropub.biology.000970

**Published:** 2023-09-17

**Authors:** Gang Wu, Ralf Baumeister, Thomas Heimbucher

**Affiliations:** 1 Signalling Research Centres BIOSS and CIBSS, University of Freiburg, 79104 Freiburg, Germany; 2 Bioinformatics and Molecular Genetics, Faculty of Biology, University of Freiburg, 79104 Freiburg, Germany; 3 Center for Biochemistry and Molecular Cell Research, Faculty of Medicine, University of Freiburg, 79104 Freiburg, Germany; 4 Faculty of Medicine, ZBMZ Center of Biochemistry and Molecular Cell Research, University of Freiburg, 79104 Freiburg, Germany; 5 FRIAS Freiburg Institute for Advanced Studies, Albertstraße 19, University of Freiburg, 79104 Freiburg, Germany

## Abstract

Maintaining iron levels is crucial for health, but iron overload has been associated with tumorigenesis. Therefore, critical enzymes involved in iron homeostasis are under tight, typically posttranslational control. In *C. elegans* , the mTORC2 and insulin/IGF-1 activated kinase SGK-1 is induced upon exogenous iron overload to couple iron storage and fat accumulation. Here we show that, already at physiological iron conditions, *sgk-1* loss-of-function increases intracellular iron levels that may impair lifespan. Reducing iron levels by diminishing cellular or mitochondrial iron import is sufficient to extend the short lifespan of *sgk-1* loss-of-function animals. Our results indicate another regulatory level of *sgk-1* in iron homeostasis via negative feedback regulation on iron transporters.

**Figure 1. SGK-1 affects SMF-1 protein- and iron levels to control lifespan:**
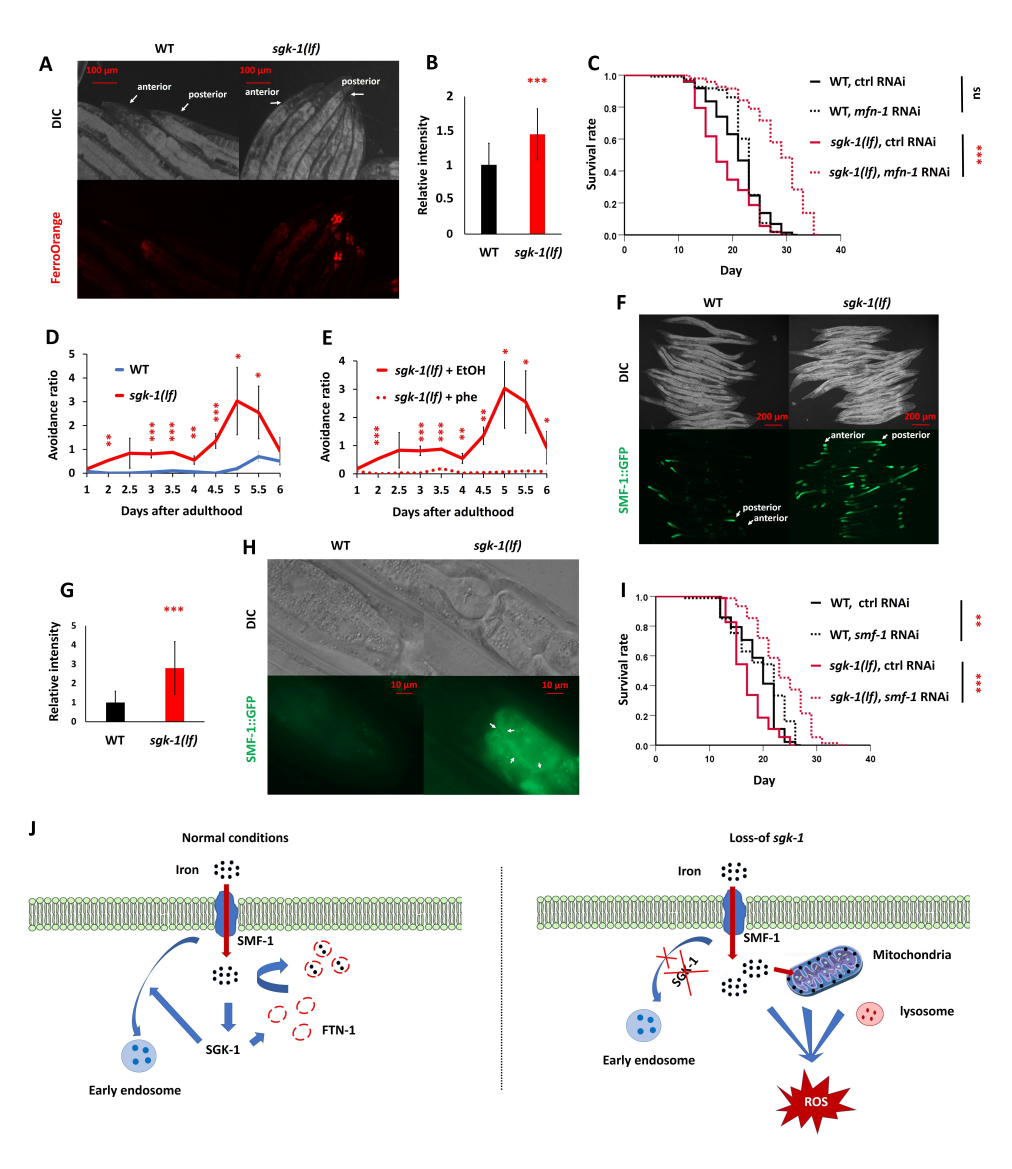
A-B: FerroOrange staining and its quantification. Adult day 5 *sgk-1 (lf)* mutants accumulate iron in the anterior and posterior intestine compared to wild type (A). 10-15 worms of each genotype were used for quantification (B). Two-tailed t-test, mean ± SD; results are representative for two biological replicates. C: RNAi-mediated knock-down of *mfn-1* (mitoferrin 1) from hatching increases lifespan in *sgk-1 (lf)* mutants. n ≥ 100. Results are representative for two biological replicates. D-E: Bacterial avoidance assay. *sgk-1 (lf)* mutants show higher bacterial avoidance from adult day 1 to day 6 compared to wild type (D), which is suppressed upon treatment with 50 µM iron chelator (phe) (E). P values were calculated using a two-tailed t-test for each time point, mean ± SD. Results are representative for two biological replicates. F-H: Adult day 1 *sgk-1 (lf)* mutants accumulate SMF-1 ::GFP in the anterior and posterior intestine. Images are taken using a 20X lens (F) and a 63X lens (H). Arrows in H indicate the apical membrane localization of SMF-1::GFP. Quantification of SMF-1::GFP fluorescence (G) from images in (F) was performed using 10-15 worms of each genotype. Two-tailed t-test for each time point, mean ± SD. Results are representative for three biological replicates. I: RNAi-mediated knock-down of *smf-1 ,* encoding a divalent cation transporter, increases lifespan in *sgk-1 (lf)* mutants. n ≥ 73. Results are representative for two biological replicates. The *sgk-1 ( ok538 ) loss-of-function* mutant was used for all experiments (A-I). *P-value <0.05, **P-value <0.01, ***P-value <0.001 for two-tailed t-test (A-I). J: Working model. SGK-1 expression is induced upon iron overload, which reduces free iron by promoting FTN-1 expression and reducing SMF-1 levels likely through facilitating SMF-1’s recycling through endocytosis. When SGK-1 function is impaired, SMF-1 might accumulate on intestinal cell membranes resulting in elevated iron uptake. Iron overload induces ROS production and mitochondrial dysfunction, which is detrimental to animals’ survival. Mitophagy might further accelerate ROS production through Fenton’s reaction by providing a low pH environment.

## Description

Iron is an essential metal vital for living cells. It has been directly associated with cell proliferation, and, in addition to many other functions, is required for mitochondrial respiration, oxygen transport via heme and DNA replication ( Abbaspour, Hurrell et al. 2014 ). Iron deficiency results in anemia and neuro-developmental and -degeneration defects ( Ndayisaba, Kaindlstorfer et al. 2019). High iron levels, in contrast, play a crucial role in breast cancer and other carcinomas ( Elliott and Head 2012).

Nutritional iron is imported across the enterocyte membrane by divalent metal transporters (DMTs) to be used within the enterocyte for iron-containing proteins, and is transported by ferrous iron transmembrane transporters like mitoferrin proteins to the mitochondria for Fe-S cluster biosynthesis ( Abbaspour, Hurrell et al. 2014). Iron not immediately utilized is stored in ferritin within the enterocyte or exported across the basolateral membrane by ferroportin ( Abbaspour, Hurrell et al. 2014).

To maintain cellular iron homeostasis within a narrow physiologically benefical range, the critical enzymes and their genes for import, transport and storage are under tight control. Several genes involved in such processes contain IRE elements (iron response elements) in transcribed sequences and are regulated posttranscriptionally by either mRNA halflife or translation initiation via mRNA-binding iron-regulatory proteins (IRPs) ( Piccinelli and Samuelsson 2007).

Almost all enzymes involved in human iron homeostasis have orthologs in the model organism *C. elegans* , which allowed to study regulatory mechanisms of iron overload on both genetic and cellular levels ( Anderson and Leibold 2014). An overload of exogenous iron was shown to induce the expression of *sgk-1* , encoding the serum-and glucocorticoid-inducible kinase 1 (ortholog of human SGK1/2/3), which in turn induced gene expression of ferritin-1 ( *ftn-1* ), vitellogenin ( *vit-2 /3* ) and a fatty acid transporter ( *acs-20* ) ( Wang, Jiang et al. 2016 ). Thus, SGK-1 synergistically regulates iron and lipid homeostasis. Several follow-up studies expanded this model to propose that SGK-1 , coupling both insulin/IGF and mTORC2 signaling, may phosphorylate and negatively regulate the zinc-finger transcription factor PQM-1 , which controls gene expression of *ftn-1* and the divalent metal transporter *smf-3* ( Dowen, Breen et al. 2016 , Rajan, Anderson et al. 2019 , Pekec, Lewandowski et al. 2022 ). However, mammalian SGK1 is better known as a regulator of ion homeostasis by controlling ion channel localization ( Lang and Shumilina 2013). This was further supported by another study in *C. elegans* , which revealed that SGK-1 regulates the intestinal endocytosis of several transmembrane proteins and therefore is also a putative regulator of additional transmembrane proteins affecting iron homeostasis ( Zhu, Wu et al. 2015).

We tested this hypothesis by first measuring intracellular iron levels in wild type and *sgk-1 (lf)* mutants under physiological conditions (no extra iron added). Surprisingly, functional loss-of *sgk-1* was sufficient to induce intracellular iron late in life, visualized via FerroOrange staining ( Fig. 1A, B ). We found that *sgk-1 (lf)* mutants have normal iron levels during adult day 1 to day 3; however, later on during adulthood, at adult day 5, they displayed increased iron levels, which is mainly detected in anterior and posterior intestinal cells ( Fig. 1A, B ). Iron overload can produce ROS through Fenton’s reaction which subsequently can directly damage DNA and proteins and produce lipid peroxide, which is detrimental to cells ( Zorov, Juhaszova et al. 2014 ). We suspected that such iron overload late in life could affect lifespan of *sgk-1 (lf)* mutants negatively. Suspiciously, there are controversial reports about *sgk-1 (lf)* mutants being either long- or short-lived ( Hertweck, Gobel et al. 2004 , Chen, Guo et al. 2013). Also in our hands, depending on nutritional food sources ( *E.coli* HT115 vs. OP50 ) and environmenal conditions, *sgk-1 (lf)* mutants’ lifespan can vary tremendously ( Mizunuma, Neumann‐Haefelin et al. 2014). Using the experimental conditions as described in our previous experiments ( Mizunuma, Neumann‐Haefelin et al. 2014), *sgk-1* loss-of-function animals’ lifespan was slightly shorter than wild type on OP50 at 20 ⁰C. Because mitochondria are the major organelle for ROS production via oxidative phosphorylation by the Fe-S containing proteins of the electron transport chain, we hypothesized that it could be the surplus of mitochondrial iron that impacts on *sgk-1 (lf)* mutants' lifespan. To test this idea, we reduced iron import into mitochondria by knocking down the mirochondrial iron importer, mitoferrin 1 ( *mfn-1* ). *mfn-1* downregulation in wild type had no effect on lifespan, but it substantially increased lifespan in *sgk-1 (lf)* beyond the lifespan of wild type animals ( Fig. 1C ). We conclude that reducing mitochondrial iron import in *sgk-1 (lf)* mutants, that have increased intracellular iron late in life, is beneficial.

This result also supports the hypothesis that *sgk-1 (lf)* animals, via iron overload, suffer from mitochondrial dysfunction. In a previous study, we had already shown that inactivation of *sgk-1* impairs mitochondrial homeostasis and triggered an increased release of mitochondria-derived ROS ( Aspernig, Heimbucher et al. 2019 ). Disruption of mitochondrial homeostasis in *C. elegans* stimulates a food avoidance behavior ( Melo and Ruvkun 2012 , Liu, Samuel et al. 2014 ). *sgk-1 (lf)* mutants, but not wild type, indeed displayed pronounced bacterial avoidance that can be fully suppressed by applying the iron chelator, 1,10-phenanthroline (phe) ( Fig. 1D, E ). We conclude that reducing mitochondrial iron levels in *sgk-1 (lf)* eliminates an avoidance behavior, which probably reflects impaired mitochondrial functions, and substantially increases lifespan. This identifies iron overload late in life as a critical aspect of loss-of *sgk-1* .

Loss-of *sgk-1* prevents expression of ferritin 1 ( *ftn-1* ), one of the major iron storage proteins in *C. elegans* ( Wang, Jiang et al. 2016 ). Thus, this may contribute to an increase in the cytosolic pool of free iron. However, given the tight control of all known aspects of iron homeostasis, we suspected that the intestinal import of iron from the gut lumen should be already negatively regulated, if intracellular iron levels increase. SGK-1 may be a candidate for the regulatory control of membrane transporters, and indeed there are many reports implying SGK-1 in ion channel localization and turnover ( Lang and Shumilina 2013 , Zhu, Wu et al. 2015 ). In *C. elegans* , SGK-1 regulates intestinal membrane trafficking and membrane protein recycling by controlling endocytosis ( Zhu, Wu et al. 2015). We reasoned that the DMT1 transporter orthologs SMF-1 /2/3 may be a target of SGK-1 regulation. DMT1 (mammalian Divalent Metal Transporter 1) is the major iron transporter responsible for cellular iron uptake, and *C. elegans* harbors three homologous genes to DMT1, *smf-1 , smf-2,* and *smf-3* . GFP fusion proteins of SMF-1 , but not SMF-3 , were increased in day 1 adult *sgk-1 (lf)* mutants ( Fig. 1F -H). Consistent with iron accumulation indicated by FerroOrange, SMF-1 ::GFP also accumulated preferably in anterior and posterior intestinal cells. This result together with SGK-1 ’s induction upon iron overload ( Wang, Jiang et al. 2016 ), suggest that SGK-1 regulates iron uptake in a negative feedback loop. If the surplus of SMF-1 is one of the causes of increased iron levels and damages in *sgk-1 (lf)* mutants, then experimenal downregulation of *smf-1* expression should be beneficial for *sgk-(lf)* animals’ lifespan. Indeed, similarly to *mfn-1* knockdown in the background of *sgk-1 (lf)* , *smf-1* RNA interference significantly increased lifespan of *sgk-(lf)* animals ( Fig. 1I ). Unlike *mfn-1* RNAi, knock-down of *smf-1* also extended longevity in wild type, indicating that already in wild type animals, when using standard laboratory growth conditions, iron concentration outside of the mitochondria may be a factor limiting lifespan.

Our results illustrate a new function of SGK-1 in a negative feedback loop in iron transporter homeostasis, that already regulates iron import into intestinal (or enterocyte) cells. Impairment of this feedback mechanism by blocking *sgk-1* activity or expression results in increased cellular iron levels ( Fig. 1J ). The production of ROS through Fenton’s reaction requires an acidic environment ( Zorov, Juhaszova et al. 2014), which is typically not found in functional mitochondria, but is acquired once mitochondria fuse with lysosomes during mitophagy. The pH requirement of Fenton’s reaction actually could explain why preventing autophagy/mitophagy, which is usually beneficial for longevity, could increase rather than further decrease lifespan of *sgk-1 (lf)* mutants ( Zhou, Kreuzer et al. 2019). In addition, our results also indicate that compromised mTORC2/ SGK-1 signaling has both positive and negative effects on lifespan; upon iron overload, its negative effect seems to dominate the positive impact of impaired mTORC2/ SGK-1 signaling. Therefore, by diminishing intracellular iron abundance, *sgk-1 (lf)* animals become long-lived compared to wild type ( Fig. 1C, I ). In consequence, dietary adjustments that reduce iron in nutrition, or alterations of the metabolome by changing the *E. coli* food sources, should also be beneficial for *C. elegans* longevity. This ambivalent effects of mTORC2/ SGK-1 signaling on lifespan, and the potential differences of iron levels of the various nematode growth media (NGM) used in different *C. elegans* laboratories, might explain the conflicting lifespan reports of *sgk-1 (lf)* mutants during the past 20 years.

## Methods

**Worm strains and maintenance**

All strains were cultured using standard methods ( Brenner 1974 ). Worm strains were maintained at 20 ⁰C on nematode growth media (NGM) plates. NGM plates were seeded with 200 µl OP50 suspension in LB (Lysogeny Broth) media one to two days before performing experiments. In all experiments, N2 is wild type.

**FerroOrange staining**

FerroOrange (Dojindo, F374-10) ( Anandhan, Dodson et al. 2023) was dissolved in DMSO to generate a 1 mM stock solution and stored at -20 ⁰C. Before staining, FerroOrange stock solution was diluted using M9 to a 10 µM working solution for staining. Synchronized adult day 5 worms were washed off NGM plates using M9 and transferred to 1.5 ml Eppendorf tubes and further washed by shaking for 5 min on a rocking shaker at 100 rpm at room temperature. Then worms were collected by centrifugation at 2000 rpm for 1 min, and supernatant was removed. Washing steps were repeated 3 times. Next, worms were stained in a 10 µM FerroOrange working solution for 1 hour, on a rocking shaker at 100 rpm at room temperature. During the staining procedure worms were protected from light by covering tubes with a paper box. After staining, staining solution was removed following centrifuation at 2000 rpm for 1 min. Then worms were washed 3 times using M9 by shaking for 5 min on a rocking shaker at 100 rpm at room temperature, also protected from light; and then worms were collected by centrifugation at 2000 rpm for 1 min to remove the supernatant. After washing, worms were mounted on a 3% agarose pad on a glass slide, using 10 mM sodium azide for immobilization of worms. Images were taken with 20 X or 63 X oil lenses using a Zeiss Axio Imager Z1 fluorescence microscope. Quantification was analyzed with image J, using the average FerroOrange signal intensity of a whole worm; and background was subtracted. The average intensity of wild-type samples was used for normalization. 10 to 15 worms were used for quantification for each condition. P values were calculated using a two-tailed t-test.

**Lifespan assay and statistics**

Worm were grown for several generations on 20 °C without starvation. To prepare synchronized worms, we transferred 20 to 25 adult day 1 to day 2 worms on freshly prepared OP50 /NGM plates and let them lay eggs for 6 to 10 hours. Adult worms were removed and larvae were grown to the young adult stage. Because the larval development of *sgk-1 (lf)* mutants is delayed by about 12 hours compared to wild type animals, the egg laying of *sgk-1 (lf)* mutants was performed 12 hours ahead of wild type. 3 days after egg laying of wild type, 100 young adult worms of each genotype were transferred to 5 new plates (with 20 worms on one plate) to initiate the lifespan assay. To prevent the bagging phenotype of *sgk-1 (lf)* mutants, FUDR was used in a final concentration of 50 µg/ml for all strains till the end of the lifespan assay. Dead worms were scored and removed every other day till all worms were dead. The log-rank (Mantel–Cox) method was used to test the null hypothesis (two-sided hypothesis test) in Kaplan–Meier survival analysis, as previously described ( Lawless 2011 ), and evaluated using the SPSS survival analysis software. All survival experiments were carried out at 20 °C; n ≥ 73 per strain/trial.

**Bacterial avoidance assay**

Around 60 synchronized young adult worms (defined as day 1) were transferred to NGM plates which were freshly seeded with OP50 a day before. Three replicates were setup for each genotype per condition. Around 10 hours after transferring worms to plates, the distribution of worms was recorded. Precisely, worms were recorded as either “inside the bacterial lawn” or “outside the bacterial lawn”. The bacterial avoidance ratio was calculated by dividing the number of worms “outside” by the number of worms “inside” the bacterial lawn. From day 1 onwards the distribution of worms was recorded twice a day, in the morning and in the evening until day 6 of adulthood. To avoid a mix of cultures with their progeny, all adult worms for the assay were transferred to new OP50 plates after the second distribution was recorded on each day. P values were calculated using a Two-tailed t-test at each time point.

**RNAi knockdown**

RNAi knockdown was performed by feeding worms with an engineered RNAi compatible OP50 bacterial strain ( *OP50 (xu363))* ( Xiao, Chun et al. 2015 ) that expresses double stranded RNA homologous to target genes for the knockdown. Recombinant plasmids containing homologous fragments for knockdown were isolated from HT115 bacterial strains from Ahringer library, and then transformed into competent *OP50 (xu363)* cells. For bacterial cultures, transformed *OP50 (xu363)* bacteria were incubated in LB media with 25 µg/ml ampicillin for plasmid selection, in a bacterial shaker overnight (37 ⁰C, 180 rpm). Bacteria were condensed 10 times before seeding onto NGM plates which contained 1 mM IPTG, 25 µg/ml ampicillin and 25 µg/ml tetracycline. For lifespan assays, worms were kept on RNAi plates from hatching till the end of the lifespan assay. Worms were transferred to new RNAi plates around day 8 and day 16 (in the presence 50 µg/ml FUDR).

**Iron chelator 1,10-phenanthroline (phe) supplementation**

1,10-phenanthroline (131377-2.5G, Merck Millipore) was dissolved in 100% ethanol to obtain a 50 mM stock solution. Aliquots of that stock solution were frozen at -20 ⁰C. To prepare NGM plates containing 50 µM phe, 50 mM phe stock was diluted 5 times in 100% ethanol to generate a 200X solution, and 70 µl 200X phe solution was distributed on the surface of seeded NGM plates (the volume of NGM plates was approximately 14 ml). For lifespan assays using phe, worms were kept from hatching on NGM plates containing either 50 µM phe or 0.5% ethanol as a control, till the end of the lifespan assay. Worms were transferred to new phe- or ethanol-containing plates around day 8 and day 16.

**SMF-1 ::GFP reporter imaging**

Synchronized adult day 1 transgenic worms expressing SMF-1 ::GFP ( Au, Benedetto et al. 2009 ), in either a wild-type or an *sgk-1 (lf)* background were used for imaging. Worms were mounted on a 3% agarose pad on a glass slide, and immobilized using 10 mM sodium azide. Images were taken with 20 X or 63 X oil lenses using a Zeiss Axio Imager Z1 fluorescence microscope. Quantification was performed with image J, using the average SMF-1 ::GFP signal intensity of a whole worm; and background was subtracted. The average intensity of wild type samples was used for normalization. 10 to 15 worms were used for quantification for each condition. P values were calculated using a two-tailed t-test.

## Reagents

**Worm strains**

**Table:** 

Strain name	Genotype	Origin
N2	wild type	CGC
BR4774	*sgk-1 ( ok538 ) *	Outcrossed in this study
MAB111	* mjaEx074[ SMF-1 ::GFP; rol-6 ( su1006 )] *	Michael Aschner lab
BR8891	*sgk-1 ( ok538 ); mjaEx074[ SMF-1 ::GFP; rol-6 ( su1006 )] *	This study
